# Elimination of *Mycoplasma* contamination in *Chlamydia* stocks as a result of in vivo passage or plaque isolation

**DOI:** 10.1186/s13104-018-3455-x

**Published:** 2018-06-07

**Authors:** Ira M. Sigar, Justin H. Schripsema, Kathleen A. Kelly, Ashlesh K. Murthy, Srikanth Manam, Kyle H. Ramsey

**Affiliations:** 1 Microbiology and Immunology Department, Chicago College of Osteopathic Medicine, Midwestern University, SH-323H, 555 31st Street, Downers Grove, IL USA; 2 Pathology and Laboratory Medicine, David Geffen School of Medicine, at UCLA, Los Angeles, CA USA; 3 College of Veterinary Medicine, Midwestern University, Glendale, AZ USA

**Keywords:** *Chlamydia*, *Mycoplasma*, Cell culture, Contamination, Plaque assay

## Abstract

**Objective:**

This study aims to eliminate *Mycoplasma* spp. contamination from laboratory stocks of *Chlamydia* spp. by in vivo passage or by plaque assay.

**Results:**

We have described two methods of eliminating *Mycoplasma* contamination from *Chlamydia* laboratory stocks. We conclude that *Mycoplasma* species commonly contaminating chlamydial stocks do not survive passage in mice. *Chlamydia* may also be derived *Mycoplasma*-free by plaque assay.

**Electronic supplementary material:**

The online version of this article (10.1186/s13104-018-3455-x) contains supplementary material, which is available to authorized users.

## Introduction

Bacteria of the class *Chlamydiae* are obligate intracellular pathogens and are commonly grown in mammalian cell lines such as McCoy, L929, or HeLa 229 cells. Unfortunately, cell culture is easily and commonly contaminated with *Mycoplasma* spp. Likewise, contamination of *Chlamydia* stocks and cultures by *Mycoplasma* spp. is common in laboratories and thus complicates interpretation of experimental results. *Mycoplasma* contamination has also been shown to confound interpretation of chlamydial serodiagnostic tests [1–4].

Detecting and confirming *Mycoplasma* in chlamydial culture can be challenging. Cultivation in broth and subsequently on agar was the long-held gold standard but can take more than 2 weeks for results. Nucleic acid detection has offered a more rapid approach to screening for *Mycoplasma* contamination, but a commercial test targeting *Mycoplasma* 16S ribosomal RNA by polymerase chain reaction (PCR) was subsequently found to cross-react due to sequence homology among *Mycoplasma* and *Chlamydia* spp. [5, 6]. A commercially available rapid test for *Mycoplasma* ATP synthase works more reliably and is both relatively sensitive and specific (MycoAlert™, Lonza, Walkersville, MD). We now routinely use this assay to screen cell cultures and chlamydial stocks for mycoplasma in our laboratory. If positive by this test, we confirm our results with PCR and primers targeting the GPO-3 (general prokaryotic oligonucleotides) and MGSO (mycoplasma genus-specific oligonucleotides) sequences [7].

A finding of *Mycoplasma* contamination is further complicated by inadequate remedies to selectively target *Mycoplasma* over *Chlamydia* in vitro. For example, antibiotics (e.g., fluoroquinolones such as ciprofloxacin) possess equally potent antimicrobial effects on all species of *Chlamydiae* in vitro (authors’ unpublished observations). Similarly, the newer anti-*Mycoplasma* compound, Plasmocin™ (InvivoGen, San Diego, California, USA) which is commercially promoted as a means to prevent or eliminate *Mycoplasma* contamination from various cell culture systems [8], is equally cidal for *Chlamydia* spp. (authors’ unpublished observations). Although a colleague recently reported to us that she was able to successfully eliminate *Mycoplasma* from *C. trachomatis*, serovar L2, and also *C. muridarum* by treatment with Mynox^®^ (Minerva Biolabs, Berlin, Germany), we have not yet verified these results (Jane C. Goodall, University of Cambridge, personal communication). With respect to this and other anti-mycoplasma treatments, we do not yet know of any that exhibit selective toxicity for *Mycoplasma* over *Chlamydia*. It is at least noteworthy that a recent report has found several *Mycoplasma* isolates resistant to each of these treatments [8]. Hence, a method for reliably and selectively targeting *Mycoplasma* spp. over *Chlamydia* spp., and thereby deriving a “pure” *Chlamydia* stock, is needed.

## Main text

### Method

#### *Chlamydia* stocks

*Chlamydia muridarum* mouse pneumonitis (MoPn) strain (Weiss), *C. trachomatis* serovar E/Bour and the *C. pneumoniae* (TW-183) were obtained and maintained by our laboratories. These stocks were positive for *Mycoplasma* contamination using the two detection methods as described below.

#### Cell lines

HeLa 229 cells were used for propagation of *C. muridarum* MoPn Weiss and *C. trachomatis* serovar E. Hep-2 cells were used for propagation of *C. pneumoniae*. The L929 cells were used for the plaque-forming assays. All cell lines are from American Type Culture Collection. Dulbecco’s modification of Eagle’s medium with 4.5 g/l glucose, l-glutamine and sodium pyruvate (Cellgro-Mediatech, Manassas, VA) supplemented with 10% fetal calf-serum and gentamicin (100 µg/ml) was used. Before infection with Chlamydial sample, the cell lines were tested by MycoAlert™ (Lonza, Walkersville, MD) to ensure that they were negative for *Mycoplasma*.

#### Animals

Female BALB/c and C3H/HeN mice were used for *C. muridarum* infection; C57BL/6 female and male mice were used for *C. trachomatis* and *C. pneumoniae* consecutively. All mice were purchased from Charles River Laboratories International (Wilmington, MA). This study was approved by the Institutional Animal Care and Use Committee of Midwestern University.

#### Decontamination procedures

Two different decontamination procedures were used in this study. The first procedure was the in vivo method of passing chlamydial stock into mice. Mice [pretreated with Medroxy-progesterone acetate (Greenstone, Peapack, NJ) 7 days before infection] were infected intravaginally with 10^5^ IFU doses of *Mycoplasma*-positive of *C. muridarum* or C*. trachomatis*. On day 4 p.i. (post inoculation) or day 7 p.i. shedding of viable chlamydiae from the lower urogenital tract was assessed by the collection of cervical-vaginal swabs. All samples were frozen at − 80 °C. Samples were thawed and expanded in HeLa 229 cell culture monolayers as previously described in Cotter et al. [9].

For *C. pneumoniae*, mice were infected intranasally with 10^5^ IFU doses of *Mycoplasma*-positive of *C. pneumoniae*. On day 4 and 7 p.i. lungs were collected, homogenized and subsequent culturing of lung tissue material in Hep-2 cell monolayers. The process of decontamination was first monitored using the MycoAlert™ detection kit. Then, the remaining sample can be passed to 6 or 12-well plates to extract DNA for *Mycoplasma* group-specific PCR assay detection and to repeat the *Mycoplasma* detection by MycoAlert™.

The second procedure of decontamination *Mycoplasma* from Chlamydial stock was plaque-forming assay as previously described [10], with minor modification. Dilutions of chlamydial stock were inoculated by centrifugation at 1100×*g* for 1 h at 37 °C onto confluent monolayer of L929 cells grown in 6-well tissue culture dishes. The infective inocula was then removed and the monolayers were overlaid with 1× DMEM, 10% FBS, 0.2% agarose, 0.2 µg/mL cyclohexamide, 50 µg/ml gentamicin, 100 µg/mL vancomycin, 0.5 µg/ml fungizone and incubated at 37 °C, 5% CO_2_ for 5–7 days. Plaque purifications were carried out by picking individual plaques into 100 µL SPG buffer then aliquots into two 50 µl suspensions and stored at − 80 °C. The suspension was used to infect individual wells of a 24-well tissue culture dish containing *Mycoplasma*-free confluent monolayers of cell line. The same procedure of monitoring decontamination was performed as described above (Mycoalert and PCR). *Note* to confirm that the plaques contain Chlamydial DNA, a PCR amplification of 16S Ribosomal *Chlamydia* gene was done as described in Wooters et al. [11], using the same template for the *Mycoplasma* group-specific PCR assay.

#### Detection of *Mycoplasma* contamination

Two methods of detecting *Mycoplasma* contamination were done to all stocks and samples. These are MycoAlert™ Mycoplasma Detection Kit and *Mycoplasma* group-specific PCR assay [5]. To use MycoAlert™ Mycoplasma Detection Kit, Chlamydial samples were infected into 24 h *Mycoplasma*-negative cell line monolayers in 24-well plates, using MOI ≤ 3. The plate was centrifuged for 1 h at 1100×*g* at 37 °C then transferred to 37 °C, 5% CO_2_ incubator. After 2 h, the media was changed to cyclohexamide media (0.5 µg/ml). Following a 24 h incubation, 500 µl samples were processed according to MycoAlert™ protocol. A sample is positive for *Mycoplasma* contamination when the ratio of B (second reading)/A (first reading) is greater than 1. Reading was accomplished using TD-20/20 luminometer Turner designs (Sunnyvale, CA) with delay time 3 s, and integration time 4 s. *Mycoplasma* group-specific PCR assay was accomplished using template from Chlamydial DNA extraction, either from high-speed purification of *Chlamydia*’s elementary bodies harvested from cell line or by scraping infected cell line directly. Extraction of DNA was accomplished using DNeasy Blood and Tissue kit (Qiagen, Valencia, CA) with 60–80 µl elution. Five microliters of sample was added to 20 µl of the following PCR mixture: 1X *Taq* PCR Master Mix (Qiagen) and 0.8 µM each primers. A PCR protocol was used in Techne TC-412 (Techne, Burlington, NJ) with 94 °C, 30 s; 55 °C, 30 s; 72 °C, 60 s for 35 amplification cycles using the primer pair of upstream primer GPO-3, 5′-GGGAGCAAACAGGATTAGATACCCT-3′; downstream primer MGSO, 5′-TGCACCATCTGTCACTCTGTTAACCTC-3′. The 270 bp of PCR product was analyzed by agarose electrophoresis.

### Results

Recently, we re-derived selected *C. muridarum* stocks following in vivo passage in mice. Several of these were known to be contaminated with *Mycoplasma* spp. (Table 1). Following inoculation in mice and subsequent in vitro expansion, the *C. muridarum* stocks were found to be negative by MycoAlert™ and PCR (initial data not shown). A more detailed analysis of additional cell culture isolates derived following in vivo passage was then conducted-where we tested 3 sets of isolates. Each isolate was derived from cervical-vaginal swabs following intravaginal inoculation of mice (*C. muridarum* or *C. trachomatis*) [12, 13]. In addition, lung homogenates of mice inoculated intra-nasally with *C. pneumoniae* [14] were assessed. Each sample listed in Table 1 was derived following an initial isolation event from mouse samples and two subsequent passages in cell culture.

**Table 1:** Effect of in vivo passage of *Chlamydia* spp. stocks on *Mycoplasma* contamination

Species (strain) route	Isolates tested (mouse strain)	Result, in vitro pass 1		Result, in vitro pass 2	
		MycoAlert™	PCR^a^	MycoAlert™	PCR^a^
*C. muridarum* (Weiss strain) intravaginal	Culture stock^b^	+	+	+^2^	+
	c-v^c^ swab 1 (BALB/c) day 4	−	−	−	nd
	c-v swab 2 (BALB/c) day 4	−	−	−	nd
	c-v swab 3 (BALB/c) day 4	−	−	−	nd
	c-v swab 4 (C3H/HeN) day 4	−	−	−	nd
	c-v swab 5 (C3H/HeN) day 4	−	−	−	nd
	c-v swab 6 (C3H/HeN) day 4	−	−	−	nd
*C. trachomatis* (serovar E/Bour) intravaginal	Culture stock^b^	+	+	+	+
	c-v swab 1 (C57BL/6) day 4	−	−	−	−
	c-v swab 2 (C57BL/6) day 4	−	−	−	−
	c-v swab 3 (C57BL/6) day 4	−	−	−	−
	c-v swab 4 (C57BL/6) day 4	−	−	−	−
	c-v swab 5 (C57BL/6) day 7	−	−	−	−
	c-v swab 6 (C57BL/6) day 7	−	−	−	−
	c-v swab 7 (C57BL/6) day 7	−	−	−	−
	c-v swab 8 (C57BL/6) day 7	−	−	−	−
*C. pneumoniae* (TW-183) intranasal	Culture stock^b^	+	+	+	+
	Lung 1 (C57BL/6) day 4	−	−	−	−
	Lung 2 (C57BL/6) day 4	−	−	−	−
	Lung 3 (C57BL/6) day 4	−	−	−	−
	Lung 4 (C57BL/6) day 4	−	−	−	−
	Lung 5 (C57BL/6) day 7	−	−	−	−
	Lung 6 (C57BL/6) day 7	−	−	−	−
	Lung 7 (C57BL/6) day 7	−	−	−	−
	Lung 8 (C57BL/6) day 7	−	−	−	−

^a^PCR for GPO-3/MGSO positive with a PCR product band at the anticipated size of ~ 270 bp on agarose gel electrophoresis. A positive control for chlamydial 16 rRNA was included in each reaction

^b^All culture stocks of each of these strains repeatedly tested positive by MycoAlert™. In this assay, a before/after ratio of ATP of 1.0 or greater is considered a positive result by the manufacturer. All of our culture stocks were positive between 5.0 and 16.0 in multiple tests for each stock

^c^c-v = cervical vaginal swab collected as previously described in Cotter et al. [9];

*nd* not determined

At each in vitro stage following in vivo sample collection, the sample was negative by MycoAlert™ and/or by PCR (Table 1). In addition, several of these samples have since been tested again on other occasions and found to be negative by MycoAlert™ and PCR (data not shown). The data infer that the strain(s) of mycoplasma contaminating multiple stocks of chlamydiae, originally derived from various sources, are not compatible with growth in the mouse host. Hence, we have demonstrated that passage through a mouse host was able to cure the chlamydial isolates of the mycoplasma contamination.

Realizing that not all laboratories are able to conduct in vivo passage to re-derive a *Mycoplasma*-free isolate, we report a second, albeit less efficient, means to rid *Chlamydia* of *Mycoplasma* contamination. Many species and strains of *Chlamydia* are known to form plaques in mouse fibroblast monolayers such as the L929 cell line [13]. Recently, when preparing clonal chlamydial isolates of *C. muridarum* for genomic sequencing, we observed that, though initially *Mycoplasma*-contaminated, several plaque isolates were *Mycoplasma*-free when expanded further in vitro. We attempted this again and found the result, while not 100% efficacious, is repeatable (Table 2). Of the 22 plaque isolates tested: (1) 11 (50%) were consistently negative for *Mycoplasma* contamination by MycoAlert™ assay and PCR following initial isolation and 2 in vitro expansion passages (plaque isolate numbers 10, 12–15 and 17–22); (2) nine were negative on MycoAlert after the first passage but were consistently PCR positive and became both MycoAlert and PCR + following the second passage (plaque isolate numbers 1–3, 5–8 and 11); (3) one isolate was only PCR + following the first in vitro passage and became negative by both assays thereafter (plaque isolate number 9); (4) one isolate was consistently positive on both assays (plaque isolate number 16) and (5) one isolate was consistently negative on MycoAlert but PCR positive on all passages (plaque isolate number 4). These results have been confirmed with further MycoAlert testing in several stocks of these isolates in subsequent passages (data not shown.)

**Table 2:** Effect of plaque isolation assay on *Mycoplasma* contamination

Plaque isolate number^a^	Result, passage 1		Result, passage 2	
	MycoAlert™	PCR	MycoAlert™	PCR
1	−^b^	+	+	+
2	−	+	+	+
3	−	+	+	+
4	−	+	−	+
5	−	+	+	+
6	−	+	+	+
7	−	+	+	+
8	−	+	+	+
9	−	+	−	−
10	−	−	−	−
11	−	+	+	+
12	−	−	−	−
13	−	−	−	−
14	−	−	−	−
15	−	−	−	−
16	+	+	+	+
17	−	−	−	−
18	−	−	−	−
19	−	−	−	−
20	−	−	−	−
21	−	−	−	−
22	−	−	−	−

^a^Stock from which random plaques were selected was *C. muridarum* (Weiss strain) and were consistently positive by MycoAlert™

^b^All culture stocks of each of these strains repeatedly tested positive by MycoAlert™. In this assay, a before/after ratio of ATP of 1.0 or greater is considered a positive result by the manufacturer. All of our culture stocks were positive between 5.0 and 16.0 in multiple tests for each stock

### Discussion

While we cannot speculate as to the origin of the *Mycoplasma* contamination of our culture stocks—whether introduced by routine cell culture passage or from the original isolation—it is safe to assert that the *Mycoplasma* strains commonly contaminating *Chlamydia* in cell culture in our lab do not survive introduction into the mouse host. This is confirmed in both the chlamydial research laboratories at Midwestern University and the University of California at Los Angeles. Furthermore, the effect was independent of mouse strain and route of inoculation. It is important to note that we cannot state whether in vivo passage will select for isolates that will exhibit disparate chlamydial phenotype(s) in vitro or in vivo when compared to the parental strain [13].

The other alternative to eliminating *Mycoplasma* contamination is to conduct plaque isolation. Although we cannot speculate what the mechanism behind these observations are, we can assert that while somewhat tedious, plaque assay may be a viable alternative to removing *Mycoplasma* contamination in laboratories that lack the ability to conduct the above-described in vivo passages. It is highly recommended to use two methods of Mycoplasma detection from the plaque, such as MycoAlert and PCR, due to the inconsistent result of this decontamination process. It should be noted, that not all *Chlamydia* spp. form plaques in mammalian cell culture (e.g., *C. pneumoniae*). Hence, this technique may not be viable for all species and strains.

In summary, we have described two methods of eliminating *Mycoplasma* contamination from *Chlamydia* laboratory stocks. Depending on the capabilities of the laboratory and the chlamydial strains being used, one or both methods should be effective. We believe the information provided will be of use to the chlamydial research community.

## Limitations

The size of study was limited due to *Mycoplasma* contamination of our *Chlamydia* stock (Additional file 1).

## Additional file

**Additional file 1.** The ARRIVE Guidelines Checklist.
